# Case Report: Case report: Administration of anticoagulant therapy after neuro-anesthesia procedure for hemorrhagic stroke patients with COVID-19 complications and its ethical and medicolegal consideration

**DOI:** 10.12688/f1000research.75630.2

**Published:** 2023-12-12

**Authors:** Taufik Suryadi, Kulsum Kulsum

**Affiliations:** 1 Ethics and Medicolegal Consultant, Faculty of Medicine, Universitas Syiah Kuala, Banda Aceh, Aceh, 23111, Indonesia; 2 Department of Forensic Medicine and Medicolegal, Faculty of Medicine, Universitas Syiah Kuala, Aceh, 23111, Indonesia; 3 Department of Forensic Medicine and Medicolegal, Dr.Zainoel Abidin Hospital, Banda Aceh, Aceh, 23126, Indonesia; 4 Neuro-anesthesia and Critical Care Consultant, Faculty of Medicine, Universitas Syiah Kuala, Banda Aceh, Aceh, 23111, Indonesia; 5 Department of Anesthesiology and Intensive Therapy, Dr.Zainoel Abidin Hospital, Banda Aceh, Aceh, 23126, Indonesia; 6 Department of Anesthesiology and Intensive Therapy, Faculty of Medicine, Universitas Syiah Kuala, Banda Aceh, Aceh, 23111, Indonesia

**Keywords:** Anticoagulants, COVID-19, Ethics and medicolegal, Hemorrhagic stroke, Neuro-anesthesia

## Abstract

**Background:**

Ethical dilemmas can occur in any situation in clinical medicine. In patients undergoing neuro-anesthesia for surgical procedure evacuation of intracerebral hemorrhage with a history of hemorrhagic stroke, anticoagulants should not be given because they can cause recurrent bleeding. Meanwhile, at the same time, the patient could also be infected with coronavirus disease 2019 (COVID-19), one of treatment is the administration of anticoagulants.

**Methods:**

A case report. A 46-year-old male patient was admitted to hospital with a loss of consciousness and was diagnosed with intracerebral hemorrhage due to a hemorrhagic stroke and was confirmed positive for COVID-19. Giving anticoagulants to patients is considered counterproductive so, an ethical dilemma arises. For this reason, a joint conference was held to obtain the best ethical and medicolegal solutions for the patient.

**Results:**

By using several methods of resolving ethical dilemmas such as basic ethical principles, supporting ethical principles, and medicolegal considerations, it was decided that the patient was not to be given anticoagulants.

**Conclusions:**

Giving anticoagulants to hemorrhagic stroke patients is dangerous even though it is beneficial for COVID-19 patients, so here the principle of risk-benefit balance is applied to patients who prioritize risk prevention rather than providing benefits. This is also supported by the *prima facie* principle by prioritizing the principle of non-maleficence rather than beneficence, the *minus malum* principle by seeking the lowest risk, and the double effect principle by making the best decision even in a slightly less favorable way as well as the medicolegal aspect by assessing patient safety and risk management.

## Introduction

A stroke is a brain disorder caused by blood vessel disorders that occur suddenly, this can be focal or global and can result in death within 24 hours of the onset of symptoms. ^1^ Strokes are the second leading cause of death worldwide, and the fourth in the United States. ^2^ Types of stroke consist of ischemic stroke and hemorrhagic stroke. ^3^ Epidemiological studies show that only about 8-18% of strokes are hemorrhagic strokes. However, a hemorrhagic stroke has a higher risk of death compared to ischemic stroke. ^1^^,^^2^ Hemorrhagic stroke can occur due to the rupture of a blood vessel in the brain. Based on the location of the bleeding, hemorrhagic stroke is divided into intracerebral hemorrhage (ICH) and subarachnoid hemorrhage (SAH). ^4^ ICH penetrates the brain parenchyma, while SAH penetrates the subarachnoid space. ^5^ Because the brain is a vital organ, if there is a hemorrhagic stroke, it can cause severe morbidity and high mortality rates. ^5^^,^^6^

ICH has been traditionally divided into two categories primary (spontaneous) and secondary. The incidence of primary ICH is 10-15%. ^2^ A primary ICH can be due to the rupture of small arteries and arterioles that have been damaged by chronic hypertension (60%), ^2^^,^^7^ and then successively caused by cerebral amyloid angiopathy (30%), ^2^ and then 10% is caused by advanced age, anticoagulation intensity, white matter disease, prior stroke, hematologic abnormalities, chronic kidney disease. ^8^ A secondary ICH is due to trauma, aneurysms and vascular malformations, vasculitis, hemorrhage conversion of infarct. ^2^ One of the main causes of ICH is hemorrhagic stroke, is spontaneous hemorrhagic stroke as it is the most severe complication of chronic hypertension. ^9^ Hemorrhagic stroke is a fatal disease and only 30% of patients survive within six months after the event. ^6^^,^^9^ Common causes of ICH are due to an aneurysm, bleeding from an arteriovenous malformation. ^2^^,^^5^ ICH is generally correlated with hypertension, anticoagulant therapy, coagulopathy, drug and alcohol abuse, neoplasms, or amyloid angiopathy. ^2^^,^^5^^,^^9^ Mortality within 30 days of the attack is 50%. ^9^ The outcome for hemorrhagic stroke is worse than for ischemic stroke with a mortality of about 10-30%. ^5^^,^^6^^,^^9^ ICH has a case fatality rate of about 40% per month and 54% per year. ^4^

There is an ethical dilemma for the administration of anticoagulants in these patients. Administration of oral anticoagulants (OAC) is considered a risk in hemorrhagic stroke because it can cause intracerebral re-bleeding, ^10^ in patients resuming OAC can increase the risk of recurrent bleeding 2.5-8%. ^11^ Enhancement use of OAC will increase the risk of bleeding. ^12^ However, in patients who are confirmed positive for coronavirus disease of 2019 (COVID-19), anticoagulation is recommended as an attempt to prevent blood clots that can worsen the patient's condition. ^12^ For patients suffering from both COVID-19 and a hemorrhagic stroke, the use of anticoagulants could be contradictory to their best interests. ^2^^,^^5^^,^^10^^,^^12^ It is necessary to solve the problem by reviewing the ethical and medicolegal aspects.

## Case report

A 46-year-old male, driver, Acehnese patient, came with decreased consciousness from two days before admission to the hospital. The patient claimed to have a headache that got worse then suddenly fell and experienced loss of consciousness. The patient denied any nausea and vomiting before loss of consciousness. When the patient was admitted to the emergency department of the hospital, an antigen swab was performed on the patient and it was declared negative for COVID-19. Examination of vital signs obtained level of consciousness with Glasgow Coma Scale (GCS) Eye-2, Motoric-5, Verbal-3 (E2M5V3), pupils were isochoric with diameter 2 mm/2 mm. Blood pressure (BP) 160/81 mmHg, heart rate (HR) 87 beat/minute, respiratory rate (RR) 20 times/minute, body temperature 36.7 ^o^C. The patient had uncontrolled stage II hypertension. He denied history of diabetes mellitus, allergies, and asthma.

Laboratory blood test results showed anemia (hemoglobin levels 8.2 gr/dl, hematocrit 24%, erythrocytes 2.8 × 10 ^6^/mm ^3^), leukocytosis (leukocytes 12.1 × 10 ^3^/mm ^3^), increased D-dimer (10570 ng/mL), platelets 299 × 10 ^6^/mm ^3^, eosinophils 3%. basophils 1%, neutrophil band 0%, segmented neutrophils 71%, lymphocytes 18%, monocytes 7%. In arterial blood gases test, it was found: respiratory alkalosis (pH 7,490 mmHg, pCO _2_ 31 mmHg, pO _2_ 110 mmHg, Bicarbonate (HCO _3_) 24 mmol/L, total CO _2_ 25 mmol/L, base excess (BE) 1.6, oxygen saturation 94%). X-ray examination of the chest revealed, the heart size was within normal limits and the lungs indicated bronchopneumonia. From the head computed tomography (CT) scan, it was shown that there was intracerebral bleeding in the basal ganglia area. Because the patient could not saturate spontaneous breathing trial, the reverse-transcription polymerase chain reaction (RT-PCR) swab was performed. The RT-PCR results confirmed the patient was positive for COVID-19. The patient was diagnosed with intracerebral hemorrhage due to hemorrhagic stroke and COVID-19 complication. The medical treatment taken was ICH evacuation craniotomy.

Neuro-anesthesia management was performed when preoperative found the patient's status was stage 3 (American Society of Anesthesiologists), ^13^ with loss of consciousness, acute increase in intracranial pressure (ICP), level of consciousness GCS E2M5V3, with history of headache and vomiting, but no history of seizures. Neurological deficit was found in the form of left hemiparesis. The patient had stage II hypertension with BP 160/90 mmHg. Cardiac examination revealed no murmur or gallop rhythm. The electrocardiography showed a sinus rhythm of 90 times/minute. The patient was obese grade II with a body mass index (BMI) of 37. ^14^ The planning that was carried out was a general anesthetic procedure with intubation, and postoperatively the patient was admitted to the intensive care unit (ICU). In the ICU, the patient had respiratory failure and was assisted with a ventilator. The patient was admitted to the ICU for 2 days, there was no improvement in the patient's level of consciousness using GCS. In this case, there was a dilemma as to whether anticoagulants could be given to hemorrhagic stroke patients with COVID-19 complications. An ethical and a medicolegal analysis is needed in making clinical decisions in these patients.

On the first day of ICU admission, the patient still experienced a decrease in consciousness with GCS of E2M5V3, BP of 159/81 mmHg, HR of 92 beats/minute, RR of 18 times/minute, and oxygen saturation of 99% (intubated). The patient was treated with intravenous fluid drip (IVFD) ringer lactate 500 cc/24 hours, head up position 30°, ceftriaxone IV 2 g/12 hours, omeprazole IV 40 mg/12 hours, phenytoin IV 100 mg/12 hours, propofol drip titration dose, fentanyl drip titration dose, amlodipine per-oral (PO) 10 mg/24 hours, and valsartan PO 160 mg/24 hours. Postoperative evaluation did not show any sign of recurrent bleeding, proven by the absence of tachycardia.

The condition of patient on the second day was similar with GCS of E2M5V3, BP of 148/61 mmHg, HR of 135 beat per minute, RR 18 times per minute, and oxygen saturation of 98% (intubated). Patient was treated by IVFD ringer lactate 500 cc/ 24 hours, head up 30° position, levofloxacin drip 750 mg/24 hours, omeprazole IV 40 mg/12 hours, phenytoin IV 100 mg/12 hours, propofol drip titration dose, fentanyl drip titration dose, Perdipine drip titration dose and paracetamol drip 1g/8 hours. There was a replacement of oral amlodipine to Perdipine drip since resistant hypertension was observed as also as the prevention of recurrent stroke and re-bleeding risks.

As the patient had severe symptoms of COVID-19, the patient was transferred to the respiratory intensive care unit (RICU) on the third day of hospital admission. The treatment remained similar from the second to eleventh day. Remdesivir 200 mg/24 hours and Combivent nebule 1 res/6 hours was added on the eight day. Tracheostomy was performed to prevent ventilator-associated pneumonia. Levofloxacin 750 mg/24 hours was replaced by meropenem 1 g/8 hours due to higher sensitivity. On the eleventh day, weaning ventilator and breathing trial was performed through tracheostomy. Clinical improvement was noticed since the twelfth day. Clinical improvement in patients can be seen from improved consciousness, patients no longer feel nauseous and vomiting, complaints of headaches disappear, patients no longer experience shortness of breath and fever, and coughing is less common.

## Discussion

### Clinical condition

In a hemorrhagic stroke common symptoms including nausea, vomiting, headache, and changes in the level of consciousness can indicate increased ICP and this is more common in hemorrhagic strokes. ^3^ Seizures are more common in hemorrhagic stroke where the incidence is up to 2-20% and is common at the onset of ICH or within the first 24 hours. ^15^ Physical examination of ICH patients in the form of changes in consciousness, shows that 30% of ICH patients are in a coma, while 28% of them have *compos mentis.* ^16^ There is a focal neurologic deficit with headache and vomiting. Upon physical examination there were also other commonly seen symptoms including moderate and hemi sensory hemiparesis deficit, lateral vision paresis, homonym, hemianopia, aphasia, positive Babinski's sign, unreactive dilated pupils. ^3^^,^^15^^,^^16^ Imaging examination in the form of a CT scan is mandatory because it is an important step in the evaluation of suspected hemorrhagic stroke, as it is used to distinguish it from ischemic stroke and can identify complications. ^3^ Magnetic resonance imaging (MRI) is also sensitive in determining bleeding but, it is difficult to perform in the acute phase. ^17^ Laboratory tests that need to be considered are prothrombin time (PT), partial thromboplastin time (PTT), international normalized ratio (INR), complete blood count (CBC), intermittent blood sugar, kidney function (ureum and creatinine), and electrolytes. ^18^

Administration of anticoagulants in patients with ICH is still controversial. Based on one study that showed medication to improve ICH outcomes, ^2^ otherwise, other studies found that the use of anticoagulants for the prevention of coronary stent thrombosis and thromboembolic stroke increased the incidence and severity of ICH. ^19^ The main problem with the use of anticoagulants is the increased risk of bleeding in general and in ICH patients in particular. ^12^ Use of warfarin in ICH can lead to hemorrhage-related death. Warfarin is thought to increase the risk of developing ICH sevenfold with a mortality rate of 60%. ^2^ If anticoagulants are given, even in small doses, to hemorrhagic stroke patients, there is a risk that bleeding will occur again, which will increase intracranial pressure which will result in decreased consciousness.

In the management of neuro-anesthesia for ICH evacuation craniotomy with COVID-19 complication, it must be ensured that there is no airway obstruction. Giving 100% O _2_ and targeted O _2_ saturation 98-100%. The respiratory rate should be around 20 breaths per minute, especially if brain herniation has occurred. Neuro-anesthesia should be ensured deep enough during intubation by endotracheal tube (ETT), so that there is no cough or increase in blood pressure that can increase intracranial pressure. Caution should be performed in patients with cervical fractures, try not to hyperextend the head, only a jaw thrust. During induction, fentanyl 200 μg and propofol 150 mg were given. The benefits of propofol in addition to induction are also useful for lowering blood pressure and intracranial pressure, followed by giving the muscle relaxant rocuronium 50 mg. Neuro-anesthesia must be ensured deep enough so that there is no increase in blood pressure fluctuations during intubation and surgery. ^2^^,^^9^^,^^16^^–^^18^^,^^20^

A rapid decrease in intracranial pressure can be achieved with the administration of diuretics. The two most commonly used diuretics are the osmotic diuretic mannitol and the loop diuretic furosemide. Mannitol is given as an intravenous bolus at a dose of 0.25-1 g/kg body weight. It is given slowly as an infusion over 10-20 minutes and is performed in conjunction with maneuvers that decrease the intracranial volume (e.g. hyperventilation). The duration of action of mannitol is 10-15 minutes and is effective for about 2 hours. ^2^^,^^9^^,^^20^ Anesthesia was maintained using a syringe pump propofol 60 mg/hour, fentanyl 50 μg/hour, rocuronium 10 mg/hour in combination with an inhalation ratio of O _2_: water: sevoflurane = 2:2: 1. Intraoperatively, it is necessary to control the intracranial pressure and try not to swell the brain further. Brain perfusion and oxygenation should be adequate. Vasopressors or epinephrine boluses should be available if needed. If possible, an invasive monitor may be placed to measure arterial blood pressure, central venous pressure, and intracranial pressure during surgery. Checking blood sugar every hour to avoid hypo or hyperglycemic. ^2^^,^^9^^,^^20^ Brain resuscitation for at least 6 hours was performed. Target O _2_ saturation was 98–100%, end-tidal CO _2_ 30 mmHg. Avoiding the occurrence of hypotension or hypertension. Balanced (zero balance), avoiding negative balance. Give oral intake via nasogastric tube (NGT) if the NGT is clear. End-tidal CO _2_ is maintained at 30-35 mmHg. Avoid hypotension and hypovolemia preoperatively, during and after surgery. ^20^ The presence of loss of consciousness with GCS E2M5V3, intracerebral hemorrhage, increased intracranial pressure, concludes that the prognosis is *dubia at malam* (doubtful tending to bad). ^2^^,^^9^^,^^20^

The incidence of hemorrhagic stroke in COVID-19 patients was 0.3% (216 of 67,155 patients) with a fatality rate of 44.72%. ^3^ COVID-19 is an infectious disease caused by severe acute respiratory syndrome coronavirus 2 (SARS-CoV-2). Infection from this virus can be transmitted by patients who are asymptomatic, pre-symptomatic, or symptomatic. COVID-19 can cause severe pneumonia, acute respiratory distress syndrome (ARDS), respiratory failure, and death. ^21^^–^^23^ As of November 18, 2021, Indonesia registered 143,709 deaths from the coronavirus. ^24^ The mortality rate from this disease is 4-5% with most deaths occur in the age group over 65 years. ^25^ The symptom varies depending on the severity of the diseases, ranging from asymptomatic patients to severe pulmonary disease with multi-organ failure. ^26^ The main symptoms are fever, cough, myalgia, shortness of breath or difficulty breathing, nausea or vomiting, and diarrhea. ^3^^,^^21^^,^^27^^,^^28^

Supporting tests consist of thorax radiography, then thorax CT-scan with contrast if needed. Pneumonia on thorax radiography imaging caused by COVID-19 would appear from normal to ground-glass opacity or consolidation. A chest CT scan can be performed to see more details of abnormalities such as ground-glass opacity, consolidation, pleural effusion, and other features of pneumonia. ^21^^,^^27^

Examination of procalcitonin will increase when a bacterial infection is suspected. Other tests are essential to see comorbidities and evaluate possible complications of pneumonia, for example, kidney function, liver function, albumin and blood gas analysis, electrolytes, blood sugar, bacterial cultures, and sensitivity tests are all used to see possible causes of bacterial infection or if a double infection with bacteria is suspected. ^18^ RT-PCR examination is a molecular examination that is often used to detect ribose-nucleic acid (RNA) that is specific for pathogenic viruses in the respiratory tract. RT-PCR examination is the gold standard in diagnosing COVID-19 due to its high sensitivity and specificity. ^21^

Management of COVID-19 patients is divided into four categories: asymptomatic, mild symptoms, moderate symptoms, and severe symptoms. In asymptomatic patients, the management of COVID-19 is self-isolation at home for 10 days after being confirmed positive. Giving non-acidic vitamin C (500 mg per 6-8 hours orally in 14 days), inhaled vitamin C (500 mg per 12 hours orally in 30 days), and multivitamins containing vitamins C, B, E and zinc (1-2 tablets per 24 hours for 30 days) are recommended. Administration of vitamin D 1000 IU or 5000 IU percutaneous and other supportive therapy are given as needed. ^21^^,^^29^

Meanwhile, COVID-19 patients with mild symptoms can be managed conservatively by self-isolation at home for 10 days after being confirmed positive and 3 days free of symptoms (fever and respiratory problems). Oral route vitamins are recommended to support the patient, such as non-acidic vitamin C 500 mg per 6-8 hours (for 14 days), inhaled vitamin C 500 mg per 12 hours (for 30 days), or multivitamins containing vitamins C, B, E and zinc 1-2 tablets per 24 hours (for 30 days). Vitamin D 1000 IU or 5000 IU percutaneous can also be given. Favipiravir with a loading dose of 1600 mg per 12 hours orally on the first day followed by a maintenance dose of 600 mg per 12 hours orally for the next 5 days. Besides, symptomatic and co-morbid therapy are given as needed. ^21^^,^^29^

COVID-19 patients with moderate symptoms are managed by hospitalization and bed rest, administration of vitamin C (200-400 mg/8 hours in 100 cc of 0.9% NaCl intravenous drip in 1 hour), and administration of vitamin D (1000 IU or 5000 IU percutaneous). Administration of chloroquine phosphate 500 mg/12 hours orally (for 5 days) or hydroxychloroquine in preparations of 200 mg or 400 mg/24 hours/oral (for 5 days), azithromycin 500 mg/24 hours/oral (for 5 days). When antiviral medicine is required, Favipiravir can be given with a loading dose of 1600 mg per 12 hours orally on the first day followed by a maintenance dose of 600mg/12 hours for the next 5 consecutive days. Remdesivir can also replace Favipiravir with initial dose of 200 mg intravenous drip on the first day followed by a maintenance dose of 100 mg/24 hours intravenous drip on the next 4-9 consecutive days. Anticoagulants such as low molecular weight heparin (LMWH) and unfractionated heparin (UFH) should only be given based on the clinical judgement of doctors whom in charge of the patient. Besides, symptomatic and co-morbid therapy are given as needed. ^21^^,^^29^

COVID-19 patients with severe symptoms are managed by hospitalization and bed rest, administration of vitamin C (200-400 mg/8 hours in 100 cc of 0.9% NaCl intravenous drip in 1 hour), administration of vitamin B (1 ampoule intravenously) and administration of vitamin D (1000 IU or 5000 IU percutaneous). Administration of chloroquine phosphate 500 mg/12 hours orally (for 5 days) or hydroxychloroquine available preparations 200 mg or 400 mg/24 hours/oral (for 5 days), azithromycin 500 mg/24 hours/oral (for 5 days). When antiviral medication is required, Favipiravir can be given with a loading dose of 1600 mg per 12 hours orally on the first day followed by a maintenance dose of 600 mg/12 hours on the next 5 consecutive days. Remdesivir can also replace Favipiravir with initial dose of 200 mg intravenous drip on the first day followed by a maintenance dose of 100 mg/24 hours intravenous drip on the next 2-10 consecutive days. Dexamethasone 6 mg/24 hours can be given intravenously for 10 days. Anticoagulants such as low molecular weight heparin (LMWH) and unfractionated heparin (UFH) should only be given based on the clinical judgement of doctors whom in charge of the patient. In critical phase with severe pulmonary symptoms, combination therapy with intravenous methylprednisolone, high-dose intravenous ascorbic acid, thiamine (vitamin B1), and low molecular weight heparin can be provided, this combination therapy is called MATH. ^27^ Besides, symptomatic and co-morbid therapy are given as needed. ^21^^,^^26^^,^^27^^,^^29^

**Figure 1.**
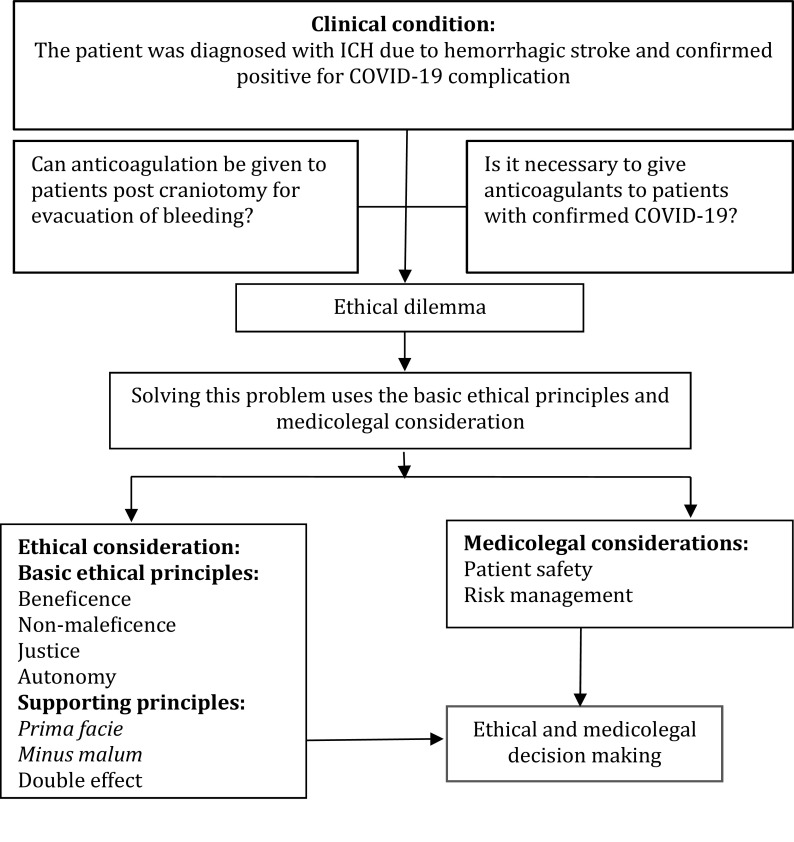
The schematic used for solving ethical dilemmas.

There is an ethical dilemma between; (a) can anticoagulation be given to patients post craniotomy for evacuation of bleeding? or (b) is it necessary to give anticoagulants to patients with confirmed COVID-19? Below, the description of solving this problem uses the basic ethical principles, namely beneficence and non-maleficence. The schematic for solving ethical dilemmas can be seen to the Figure 1. Therefore, ethical and medicolegal considerations in hemorrhagic stroke patients with COVID-19 complications should be carried out by reviewing several ethical and medicolegal principles such as *prima facie*, *minus malum*, double effect, patient safety, and risks management.

### Ethical dilemma resolution

The decision to administer anticoagulation in hemorrhagic stroke patients with confirmed COVID-19 with moderate to severe symptoms using basic ethical principles is still a dilemma. However, using basic ethical principles and other supporting principles can help resolve this ethical dilemma. ^30^ Ethical decision making should be performed by balancing all ethical principles, whether beneficence with non-maleficence, autonomy with justice, non-maleficence with justice or others. Of course, the best decision is that if all the principles can support each other, but it is possible that one of these ethical principles contradicts another, in this instance the choice is determined by which ethical principle at that time has the strongest moral justification. ^31^^,^^32^ In cases as complex as this case study, an ethical dilemma arises, then the doctor can apply *prima facie* principles among the four basic ethical principles above in Figure 1 to make ethical decisions. ^30^^,^^33^^,^^34^ In applying the *prima facie* principle, a valid new context is needed for the patient or family at the time of medical treatment (medical decision making can change if there is a more appropriate context. For example, in this case not giving anticoagulants, is a more appropriate context as preventing harm is more important than providing benefits). In this context, non-maleficence beats beneficence, in other situations it could be in that beneficence changes to non-maleficence. ^30^^–^^32^

The beneficence principle aims to provide maximum benefit for the patient while balancing benefits and risks. ^30^^,^^31^ The principle of beneficence is defined as the obligation of doctors to provide actions that are beneficial to patients. This principle will support several moral rules to protect patient rights such as to prevent harm and save patients from harm. ^30^^,^^33^^,^^34^ According to the previous discussion, patients who are confirmed positive for COVID-19 need to be given anticoagulants because of the high risk of blood coagulation. ^10^^–^^12^^,^^22^ With the provision of anticoagulants, it is expected that the disease burden of COVID-19 patients will be reduced so that the patient's body can resist infection with the SARS-CoV-2 virus. ^22^ Aspects of the principle of beneficence are expected to lead to an improvement in the patient's condition. ^30^^,^^32^^–^^34^

The principle of non-maleficence is that any medical service should not harm the patient. ^30^^,^^31^ The principle of non-maleficence stems from the doctor's obligation not to harm the patient. ^30^^,^^32^^–^^34^ In this case, the administration of anticoagulants to patients who have previously suffered intracranial bleeding are at risk for recurrent bleeding. ^6^ This principle focuses on not hurting or reducing the ability of the patient. ^31^^,^^32^ Therefore, doctors must always consider the benefits and risks that will be felt by the patient. ^30^^,^^31^^,^^33^ By using the principle of non-maleficence, it means that the doctor must consider the harm that will be experienced by the patient, either directly or indirectly, ^30^^,^^31^ when given anticoagulants.

However, it is important to understand that solving ethical dilemmas is not always easy. ^33^^,^^34^ There are times when we have to make complicated decisions by choosing which of the two conditions is the most important. ^30^^,^^32^^,^^33^ For example, in this case where giving anticoagulants is dangerous for hemorrhagic stroke patients but if they are not given, there is a risk for patients with COVID-19, here the doctor must choose between them. In this condition, the *minus malum* principle can be used, the *minus malum* principle is making decisions by choosing the least risk or harm. ^30^^–^^32^ In this patient it was decided not to be given anticoagulants because the patient had just undergone ICH evacuation surgery. Treating the patient with anti-coagulants could cause re-bleeding endangering the patient. No anticoagulation was planned for a while until there was no longer a potential for recurrent bleeding. Delaying anti-coagulant administration is certainly also a risk for COVID-19 patients because it can cause thrombosis which has the potential to become ARDS. Both of these risks are bad, but the doctor should make what they feel is the right decision, namely choosing the lightest risk.

The administration of anticoagulants in this case has a double effect, one of which is treatment for COVID-19 patients but is contraindicated in hemorrhagic stroke patients. ^34^^,^^35^ Not giving anticoagulants to these patients is certainly not a good deed when viewed from the context of handling COVID-19, but of course the goal is the health and safety of the patient regards to avoiding ICH re-bleeding. In such conditions, the principle of double effects can be used. The principle of double effect is decision making where good goals can only be done in a bad way. The double effect principle supports the principle of non-maleficence. ^33^^–^^35^ The use of *prima facie*, double effect and *minus malum* principles can be used together in synergy to make it easier for doctors to make medical decisions. In this principle, the action taken is the maximum benefit for the patient and the least risk of harm that may be caused. ^30^^–^^33^

Determination of medical indications in hemorrhagic stroke patients can be done using the principles of beneficence and non-maleficence. The principle of beneficence means that therapy must provide medical benefits, while non-maleficence means that it must not harm the patient medically. ^30^^,^^31^^,^^34^ Measurement of quality of life is determined using the principles of beneficence, non-maleficence and autonomy. ^33^^,^^34^ Quality of life is a form of satisfaction, value statement, experienced in all aspects good or bad. The quality of life of a post neuro-anesthesia patient needs to be considered because the condition of pre-anesthesia patients is already bad. In this condition, ethical considerations can be created with a *prima facie* approach by prioritizing the interests of the patient or with a *minus malum* approach by choosing a more minimal loss or with the principle of double effects. ^30^^,^^32^^,^^34^^,^^35^

### Medicolegal consideration

According to the explanation of Article 43 of the Indonesian Health Law number 36 of 2009 what is meant by patient safety is a process in a hospital that provides safer patient services. ^36^ This includes risk assessment, identification and management of patient risks, incident reporting and analysis, the ability to learn and follow up on incidents, and implement solutions to reduce and minimize risks. ^36^^–^^38^ Risk management in neuro-anesthesia includes preventive measures and management evaluations that have been carried out to reduce morbidity and mortality. ^9^^,^^37^ Risk management is an effort that tends to be proactive and can also be an evaluation of previous experience to be applied in an effort to reduce or prevent similar problems in the future. ^38^ There are four risk management steps in neuro-anesthesia: (1) problem detection (2) problem assessment (3) problem resolution, and (4) verification. ^37^

The first step in risk management is to detect the problem, in this case, patients with hemorrhagic stroke with intracerebral bleeding have an increased risk of recurrent bleeding when given anticoagulants. ^10^^–^^12^ The second step is to assess the problem, the problem experienced by the patient is that when the patient has finished the intracerebral bleeding evacuation operation, the patient has respiratory failure, so an RT-PCR swab examination is found and confirmed positive for COVID-19. This means patients would benefit from anticoagulants to reduce COVID-19 symptoms. ^26^^,^^27^ Here there is a dilemma between anticoagulants being given or not given considering the contradictory clinical condition of the patient. The third step, solving the problem, in this case it the solution to the problem was to prevent re-bleeding so no anticoagulants were given for a while. Meanwhile, to reduce the symptoms of COVID-19, airway management is carried out in the form of tracheal intubation. ^26^^,^^39^ The fourth step is verification, perioperative neuro-anesthesia management starting from the emergency room, in the operating room and in the ICU also affects the outcome of postoperative patient conditions. ^40^^-^^42^

To reduce postoperative risk, ETT removal was not performed, the patient had a respiratory failure due to COVID-19 symptoms. In this case, as in most neurosurgery patients, the patient was awakened from the effects of anesthesia as soon as possible, so that the neurological status can be evaluated as soon as possible due to surgery. ^39^ This is also in accordance with the procedure that in patients with intracerebral bleeding, complications or potential for recurrent bleeding, ETT removal is not performed immediately which is often referred to as slow weaning or delayed ETT removal. Delayed ETT removal can be performed on the conditions: poor preoperative level of consciousness, risk of edema or increased edema such as prolonged surgery, heavy bleeding, near vital areas, extensive surgery, and preoperative difficult airway management. ^42^^,^^43^

Risk management of neuro-anesthesia procedures in hemorrhagic stroke patients focused on early resuscitation, hemodynamic stabilization, and emergency surgery to evacuate bleeding. Surgery is performed by keeping the brain relaxed (by giving adequate relaxants, adequate analgesics, normal body fluid volume, and maintaining hemodynamics), lowering cerebral blood flow (CBF) thus ICP is low, protecting nerve tissue from ischemia and injury, maintaining cerebral perfusion pressure (CPP), reduce cerebral metabolic oxygen level (CMRO _2_), and optimize brain oxygen delivery (DO _2_). ^41^^–^^43^ Hemorrhagic stroke is a big matter in the medical practice due to the high mortality and morbidity. There are three goals of the anesthesiologist besides facilitating surgery, namely (1) controlling intracranial pressure and brain volume, (2) protecting nerve tissue from ischemia and injury,(3) reducing bleeding. ^9^^,^^42^

## Conclusions

By using the basic ethical principles which are assisted by the medicolegal principles, it is concluded that in hemorrhagic stroke patients with COVID-19 complications are not given anticoagulants considering the ethical principles of beneficence and non-maleficence and are supported by *prima facie* principles, double effect and *minus malum* as well as medicolegal aspects by examining patient safety and risks management. Giving anticoagulants is controversial because it can harm the patient (non-maleficence principle), although it is beneficial for patients with COVID-19 (beneficence principle), in this case, preventing harm is of higher than providing benefits. The *prima facie* principle in this case, is that beneficence turns into non-maleficence. Based on the *minus malum* principle, in this case the smallest risk was chosen, namely not giving anticoagulants to a patient with COVID-19 to prevent a greater risk of re-bleeding. The principle of double effect is carried out for the same reason, namely doing bad deeds (not giving anticoagulants) with good intentions (preventing harm to the patient if re-bleeding occurs). Risk management in patients similar to this case is to minimize the risks that occur for overall patient safety.

## Recommendations

In making medical decisions, it should be based on ethical decision, both in dilemma and non-dilemma cases. Medical decision making based on ethical decisions will add value to these decisions both morally, medically and professionally.

## Data availability

All data underlying the results are available as part of the article and no additional source data are required.

## Patient perspective

After discussing with the medical team in charge of the patient, the family was conscious of the medical reasoning and agreed to the doctor's clinical consideration of not giving anticoagulants for a certain duration for the sake of patient safety. After several days of admission in ICU and RICU, clinical improvement was noticed, hence the patient was discharged from hospital followed by outpatient management.

## Consent

Written informed consent for publication of their clinical details and or clinical images was obtained from the son of the patient because when we discussed this case, the patient was unconscious.

## Author contribution

**Suryadi T:** Project Administration, Writing-Original Draft Preparation, Writing-Review and Editing **; Kulsum K:** Supervision, Validation, and Visualization.
