# Supplementary material for: Case Report: Case report: Administration of anticoagulant therapy after neuro-anesthesia procedure for hemorrhagic stroke patients with COVID-19 complications and its ethical and medicolegal consideration
Source: F1000Res. 2023 Dec 12;10:1303. Originally published 2021 Dec 22. [Version 2] doi: 10.12688/f1000research.75630.2 (PMC10739180; doi:10.12688/f1000research.75630.2)
Supplement: Peer review history

# Reviewer report: Reviewer response for version 2

**DOI:** 10.5256/f1000research.159891.r229534

**Published:** 2023-12-21

**Authors:** Aladeen Alloubani

**Affiliations:** 1 Nursing Research Unit, King Hussein Cancer Center, Amman, Jordan

The authors responded to all my comments. However, I have a couple of comments.

1. While the ethical considerations in the study are thoroughly addressed, further exploration of the ethical decision-making process could add depth. This may include examining alternative viewpoints or incorporating additional ethical principles that might apply to the case. Such an expansion would provide a richer understanding of the complexities involved in making medical decisions under challenging circumstances.
2. The study would benefit from including more detailed descriptions of the patients' and families' perspectives. Understanding their perceptions of the treatment plan and their roles in the decision-making process would provide valuable context. This addition could enrich the discussion of ethical considerations by highlighting the human element in medical decision-making, especially in complex cases like these.

Are enough details provided of any physical examination and diagnostic tests, treatment given and outcomes?

Yes

Is the case presented with sufficient detail to be useful for other practitioners?

Yes

Is sufficient discussion included of the importance of the findings and their relevance to future understanding of disease processes, diagnosis or treatment?

Yes

Is the background of the case’s history and progression described in sufficient detail?

Yes

Reviewer Expertise:

NA

I confirm that I have read this submission and believe that I have an appropriate level of expertise to confirm that it is of an acceptable scientific standard.

# Reviewer report: Reviewer response for version 1

**DOI:** 10.5256/f1000research.79524.r220572

**Published:** 2023-11-15

**Authors:** Aldy Safruddin Rambe

**Affiliations:** 1 Department of Neurology, Faculty of Medicine, Universitas Sumatera Utara, Medan, Indonesia

The report was quite straightforward and clear, except for the last part where the author stated "Clinical improvement was noticed since the twelfth day." It would be more helpful to explain what clinical improvement was shown (ex; did the level of consciousness got better? etc.,).

 The author also stated that the patient got better without the use of anticoagulant to treat the patient's COVID-19. It would be better to explain the use of the medication in this condition in the discussion section, what could be the effect of the lack of use. On the other hand, there's sufficient reasoning about the lack of use of said medication, that is good.

Are enough details provided of any physical examination and diagnostic tests, treatment given and outcomes?

Partly

Is the case presented with sufficient detail to be useful for other practitioners?

Yes

Is sufficient discussion included of the importance of the findings and their relevance to future understanding of disease processes, diagnosis or treatment?

Yes

Is the background of the case’s history and progression described in sufficient detail?

Yes

Reviewer Expertise:

Neurovascular, Neurology

I confirm that I have read this submission and believe that I have an appropriate level of expertise to confirm that it is of an acceptable scientific standard.

# Reviewer report: Reviewer response for version 1

**DOI:** 10.5256/f1000research.79524.r118436

**Published:** 2022-02-08

**Authors:** Aladeen Alloubani

**Affiliations:** 1 Nursing Research Unit, King Hussein Cancer Center, Amman, Jordan

The current case report discussed an ethical dilemma which can occur in any situation in clinical medicine. In patients undergoing neuro-anesthesia for the surgical procedure evacuation of intracerebral hemorrhage with a history of hemorrhagic stroke, anticoagulants should not be given because they can cause recurrent bleeding. Meanwhile, at the same time, the patient could also be infected with coronavirus disease 2019 (COVID-19), for which one of treatments is the administration of anticoagulants.

 Title: The case report was repeated twice in the title. So, please delete one.

 Page 3: I suggest deleting the name of the hospital "Zainoel Abidin Hospital" to maintain privacy.

 Page 5 and 6: Need to provide citation support for all statements of fact that are not common knowledge. Would you please review and revise throughout the document?

 Page 8: I advise adding a recommendation section.

Are enough details provided of any physical examination and diagnostic tests, treatment given and outcomes?

Yes

Is the case presented with sufficient detail to be useful for other practitioners?

Yes

Is sufficient discussion included of the importance of the findings and their relevance to future understanding of disease processes, diagnosis or treatment?

Yes

Is the background of the case’s history and progression described in sufficient detail?

Yes

Reviewer Expertise:

NA

I confirm that I have read this submission and believe that I have an appropriate level of expertise to confirm that it is of an acceptable scientific standard, however I have significant reservations, as outlined above.

# Response

**Published:** 2022-07-18

**Authors:** Taufik Suryadi

**Affiliations:** Universitas Syiah Kuala, Indonesia

Dear Dr Aladeen Alloubani,

 Thank you for the excellent comments and suggestions for improving this manuscript

 Regarding the title that contains the word "case report" twice, yes, we agree to delete one of them. We request the editor to remove it from the publication manuscript.

 Page 3: Yes we agree to remove the name of the hospital "Zainoel Abidin hospital" to maintain privacy. We request the editor to remove the hospital's name from the publication manuscript.

 Page 5 and 6: We have compiled reading sources from the latest references that we found including the COVID-19 management guidelines that apply in our country (Indonesia).

 Page 8: We add recommendations namely: 

 In making medical decisions, it should be based on ethical decisions, both in dilemma and non-dilemma cases. Medical decision making based on ethical decisions will add value to these decisions both morally, medically and professionally.
